# Microbiome Engineering in Dairy Cattle: A Critical Review of Strategies for Disease Resistance, Productivity, and Sustainable Farming

**DOI:** 10.3390/vetsci13080766

**Published:** 2026-07-31

**Authors:** Muhammad Shahzaib Ashfaq, Mohamed Tharwat, Sohail Ahmed, Aftab Shaukat, Nourhan Nassar, Samavia Abid, Muhammad Ahmad Abid, Fahad A. Alshanbari

**Affiliations:** 1 Faculty of Veterinary and Animal Sciences, University of Poonch Rawalakot, Azad Jammu & Kashmir 12350, Pakistan; dr.sohail005@gmail.com (S.A.); samaviaabid11@gmail.com (S.A.); drahmadabid@gmail.com (M.A.A.); 2 Department of Clinical Sciences, College of Veterinary Medicine, Qassim University, P.O. Box 6622, Buraidah 51452, Saudi Arabia; atieh@qu.edu.sa; 3 Animal Science Division, Department of Modern Agriculture, College of Life and Environmental Sciences, Hunan University of Arts and Science, Changde 415000, China; dr.aftabshaukat@huas.edu.cn; 4 Department of Clinical Pathology, Faculty of Veterinary Medicine, Benha University, Moshtohor 13736, Egypt; nourhanadel3434@gmail.com; 5 Department of Medical Biosciences, College of Veterinary Medicine, Qassim University, P.O. Box 6622, Buraidah 51452, Saudi Arabia

**Keywords:** CRISPR, rumen microbiota, dairy cattle, probiotics, methane emissions, antimicrobial resistance

## Abstract

Dairy production currently faces three converging challenges: the escalation of antimicrobial resistance (AMR), rising global food demand, and stricter regulatory requirements for reducing enteric methane emissions. This review evaluates probiotics, prebiotics, fecal microbiota transplantation (FMT), metagenomic tools, and Clustered Regularly Interspaced Short Palindromic Repeats (CRISPR)-based synthetic biology for dairy cow microbiome engineering, applying a Technology Readiness Level (TRL) 1–9 framework to assess the translational maturity of each strategy. The quantitative ranges below are from individual controlled or field studies unless indicated otherwise, and they represent the variation from study to study in different breeds, feeds, and stages of lactation, as well as in management systems. A systematic literature search was conducted across five major databases for the period 2020–2026. Applying the TRL framework revealed that conventional probiotics have reached field-ready maturity (TRL 7–8), boosting milk yield by 0.5–1.5 kg/d and lowering somatic cell counts by 20–40%. Calf gut maturation was found to be two to three weeks faster when FMT was used (TRL 5–6). Controlled conditions (TRL 2–3) showed a 10–20% reduction in methane emissions using engineered rumen bacteria (CRISPR). Intervention failures primarily stem from host-microbiome misalignment rather than microbial product design. The key translational gap is shifting from uniform herd-level to precision-guided individualized dosing. Standardized data infrastructure, regulatory frameworks for engineered biologics, and integration with precision livestock farming platforms are required to reduce antibiotic use and lower methane emissions within a One Health framework.

## Simple Summary

Dairy farming faces growing challenges as farmers must produce more milk while reducing disease, lowering the use of antibiotics, and limiting environmental impacts. This review examines current and emerging ways to improve the natural communities of helpful microorganisms that live inside dairy cows to support better health and productivity. It compares different approaches, including beneficial feed supplements, the transfer of healthy gut microorganisms, advanced gene-based methods, and data-guided farm management, to assess how close they are to routine use on farms. The evidence shows that beneficial feed supplements are already improving milk production, supporting animal health, reducing disease, and lowering the need for antibiotics. Other promising methods have shown encouraging results but still require further research before they can be safely applied on a large scale. Overall, the review concludes that future progress will depend on choosing the most suitable approach for each individual animal rather than using the same solution for every herd. These advances could help create healthier dairy cows, more sustainable farming systems, safer food production, and a cleaner environment.

## 1. Introduction

Traditional dairy herd management relies on a combination of balanced nutrition, biosecurity and hygiene practices, vaccination, reproductive management, appropriate housing, routine health monitoring, and antibiotic treatment when clinically indicated [1]. These methods were satisfactory under the current production conditions; however, their drawbacks have become more evident. By 2050, the world’s population is expected to be 9.7 billion, with significantly higher demands for dairy products. Simultaneously, AMR (Antimicrobial Resistance) is becoming a major problem across food-producing systems, enteric methane emission from livestock, which is estimated to rise from 6.2 to 9.1 billion tonnes CO_2_ by 2050 without any extra mitigation measures, is increasingly subject to very strict regulations, and continues to limit agricultural intensification due to soil degradation [2,3].

Over the past 15 years, the study of microbial ecology has revolutionized our understanding of dairy cows as complex host-microbiome systems. Microbial communities in the rumen, gastrointestinal tract, udder, and skin play a significant role in the nutrition, immunity, and homeostasis of the host. These communities modulate fermentation efficiency, drive inflammatory responses, and regulate susceptibility to metabolic and infectious diseases in response to changes in diet, antibiotic administration, parturition, and thermal stress. Consequently, microbial imbalance is directly associated with increased incidence of mastitis, reduced milk yield, and impaired reproductive function [4]. Microbiome engineering encompasses diverse strategies to intentionally modify the bovine microbiome to enhance host health and productivity in livestock. The use of probiotics and prebiotics is an established strategy. CRISPR-modified rumen microorganisms, synthetic microbial consortia, FMT (fecal microbiota transplantation), and machine-learning-assisted metagenomic analysis are recent developments [5,6].

This review takes the stance that most failures of microbiome manipulations in dairy cattle do not stem from fundamentally inadequate microbial product design alone, but rather from imprecise matching of interventions to the baseline microbiome, farm environment, and individual animal management. No previous review has classified strategies for engineering the microbiome of dairy cattle, from laboratory proof of concept to dairy farm application, using a Technology Readiness Level (TRL) framework. This review aims to bridge this gap by summarizing the available evidence, highlighting critical evidence gaps, and outlining a roadmap for translating precision microbiome management into dairy production.

## 2. Materials and Methods

This review was conducted using a systematic literature search in accordance with the Preferred Reporting Items for Systematic Reviews and Meta-Analyses (PRISMA) 2020 guidelines; a formal meta-analysis was not performed. But synthesized the available evidence using a narrative approach rather than a quantitative meta-analysis. Throughout the manuscript, the terms systematic search and narrative synthesis are used consistently. Studies relevant to our study were identified in PubMed, Scopus, Web of Science, Google Scholar, and CAB Abstracts, and were published as peer-reviewed articles between 2020 and 2026. Although the systematic search was restricted to studies published between 2020 and 2026, a limited number of earlier references were included because they were identified through backward citation searching or represented foundational studies describing established concepts, methodological frameworks, or biological mechanisms. These references were cited for background context and were not used as primary evidence in the qualitative synthesis. All databases were last searched on 15th May 2026. The following search terms were used alone and in combination: dairy cattle microbiome, rumen microbiota, probiotics in dairy cattle, fecal microbiota transplantation in ruminants, CRISPR in rumen microbes, and precision livestock farming. The full search strategy combined controlled vocabulary and free-text terms using Boolean operators. The complete search strings for each database are provided in Supplementary Table S1.

Articles had to involve original research or a peer-reviewed review of dairy cattle microbiomes and provide an evaluation of at least one of the following: milk production, feed efficiency, methane production, or incidence of disease. Full-text availability in English was also required. Articles were excluded if they were performed only in nonruminant laboratory animals, were only conducted on human microbiomes and not directly relevant to bovine biology or were only available as conference abstracts without any accompanying peer-reviewed article. In addition to the primary outcomes described above, data were extracted on study design, sample size, animal breed and production stage, intervention type and dosage, geographic location, and funding source, where reported. Titles and abstracts were screened independently by two reviewers. Inter-reviewer agreement was not quantified using a statistical measure such as Cohen’s κ; instead, all disagreements were resolved through discussion until consensus was achieved. However, the lack of a quantitative agreement statistic is a drawback, since it is not possible to evaluate the reliability of screening independently by the readers. The study selection process, including identification, screening, eligibility assessment, and final inclusion of studies, is summarized in the PRISMA 2020 flow diagram (Supplementary Figure S1).

Each microbiome engineering strategy was independently assigned a TRL score on a 1–9 scale by two reviewers using predefined criteria. TRL 1–2 was awarded if the basic principle was observed in vitro or in silico. TRL 3–4 was applied to the confirmed proof-of-concept in a controlled animal model. If the technology was validated using pilot farm or small-scale field data, TRL 5–6 was assigned. TRL 7–8 was applied, where a successful application was demonstrated on an operational farm with multiple herd trials. TRL 9 was only for full commercial deployment purposes. In cases where the reviewers’ ratings differed by more than one level, the ratings were negotiated through structured discussions among the reviewers. Representative examples were used to ensure consistent TRL assignment across all strategies. Multiple independent multi-herd field trials and the long history of commercial application of probiotics on dairy farms classified them as TRL 7–8. Due to the limited evidence from pilot-farm and small-scale field studies, not conducted with standardized regulatory procedures, FMT was assigned TRL5–6. CRISPR-based approaches were considered TRL 2–3 as all known studies were conducted under controlled laboratory or animal model conditions, and no field-scale or commercial use of the technologies had been reported. All the strategies presented in Table 2 were followed with the same set of criteria. The TRL framework was used as an analytical tool to characterize the stage of translational maturity rather than as a scoring tool. As this review followed a narrative approach, a formal study-level risk-of-bias assessment was not conducted. Since the review used a TRL-based synthesis rather than a statistical meta-analysis, data conversion and management of missing summary statistics were not required. When studies reported different quantitative outcomes, these variations were examined qualitatively based on factors such as animal breed, dietary background, lactation stage, and intervention protocol (Section 5.1 and Section 9). Formal sensitivity analysis was not applicable because no quantitative meta-analysis was performed.

There are some methodological limitations of this review. The search was restricted to the five databases and English-language publications, which may have meant that sources in other languages or gray literature were not accessed. Study-level risk of bias (e.g., ROBIS or AMSTAR 2 or Newcastle–Ottawa Scale), quantitative meta-analysis, and certainty-of-evidence grading (e.g., GRADE) were not conducted. These approaches were also considered but rejected as they included a variety of in vitro experiments, controlled animal studies, field observations, and review articles that do not have a single validated assessment tool. Studies were therefore synthesized without methodological weighting, and ranges of quantitative findings in Section 5, Section 6, Section 7, Section 8, Section 9 and Section 10 should be taken as summaries of findings from the included studies, and not as pooled or quality-weighted estimates. Thus, the results presented should be interpreted as a synthesis of the evidence in the narrative, not as a combined effect estimate from statistical methods. Moreover, the review was also not registered in advance, potentially undermining the transparency of the pre-planned goals and benefits. The reporting of bias was not formally evaluated (e.g., funnel plot analysis) due to the narrative design and the diversity of outcome measures across studies. Because preparation of the manuscript extended beyond the final database search conducted on 15 May 2026, an additional hand-search of PubMed and Google Scholar was performed in July 2026 to identify any major developments in in vivo CRISPR applications or large-scale bovine FMT field trials. No newly published evidence was identified that would warrant revision of the TRL classifications presented in this review. The additional literature remained consistent with the pre-commercial (TRL 2–3) status of CRISPR-based approaches and the protocol-dependent (TRL 5–6) status assigned to FMT.

## 3. The Dairy Cattle Microbiome: Composition and Function

The microbiome is a complex ecosystem of commensal, symbiotic, and pathogenic microorganisms that inhabit a well-defined biological niche [7]. Although volatile fatty acids (VFAs) and short-chain fatty acids (SCFAs) refer to the same group of metabolites, including acetate, propionate, and butyrate, the two terms are conventionally distinguished in ruminant biology according to their anatomical site of production rather than their chemical structure. Throughout this review, VFAs refer to fermentation products generated in the rumen, where they represent the principal energy source for the host, whereas SCFAs refer to the same metabolites produced in the lower gastrointestinal tract, where they primarily contribute to mucosal signaling, gut barrier function, and local immune regulation. This terminology is used consistently throughout the manuscript. Endotoxins are lipopolysaccharide (LPS) molecules released during the lysis of Gram-negative bacteria, which can cause systemic inflammation if they enter the bloodstream. Table 1 lists the main body sites, dominant microbial species, primary functions, and quantities of health importance in dairy cows.

**Figure 1:**
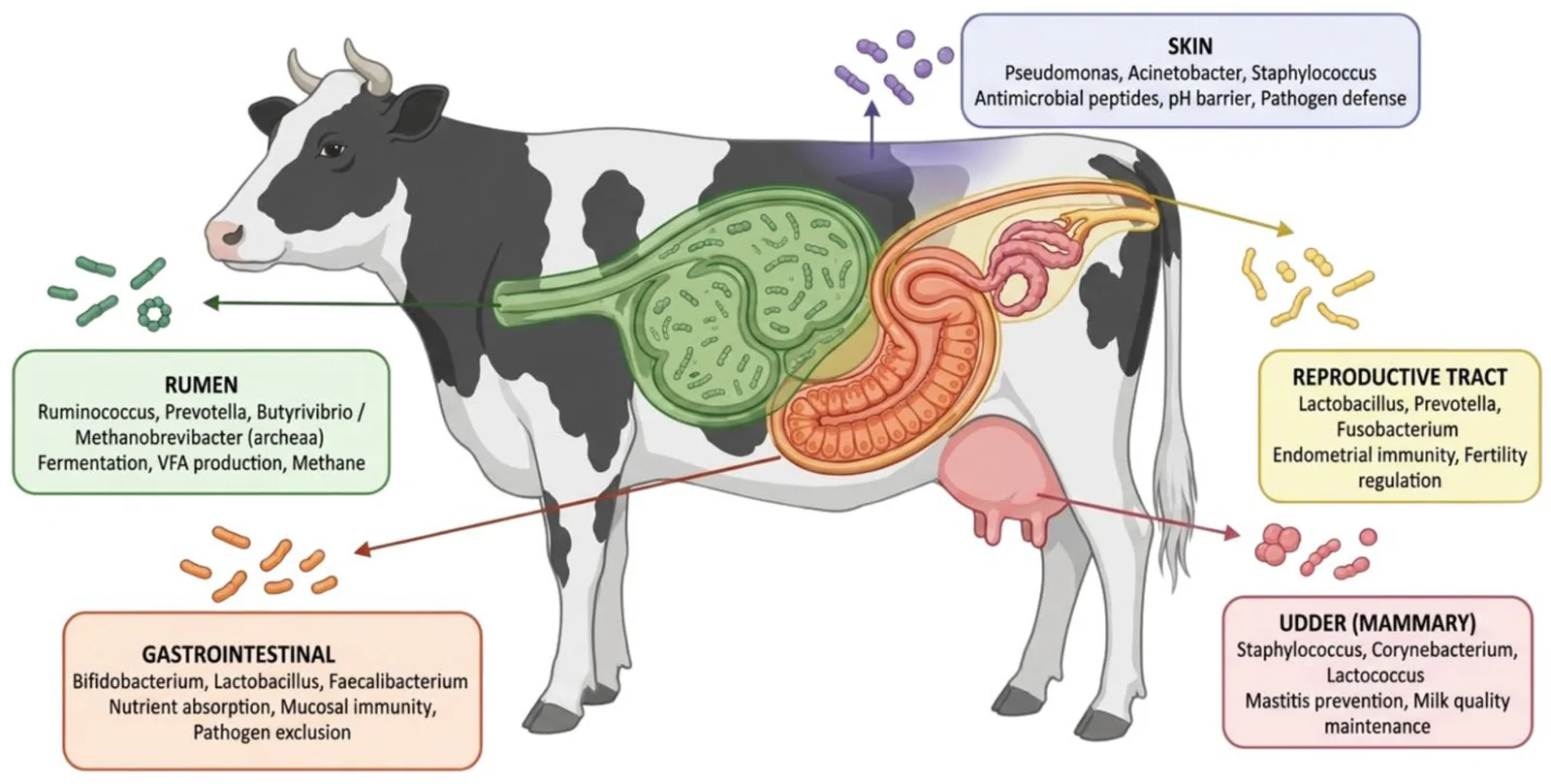
Anatomical distribution of the bovine microbiome showing representative microbial taxa and functional contributions across the rumen, gastrointestinal tract, udder, and skin, with systemic inter-niche communication via immune and metabolic signaling pathways. Created in BioRender. Ashfaq, S. (2026); https://BioRender.com/p7u2jto.

The rumen is the most important functional microbial niche in dairy cows. Plant fiber is broken down by bacteria, archaea, protozoa, and fungi into VFAs, which are the main source of energy for the host [9]. The lower gastrointestinal tract is important for nutrient absorption and mucosal immune defense and is the site of colonization resistance against opportunistic pathogens [10]. The mammary microbiome is relatively less diverse and is linked to susceptibility to mastitis and high SCC in milk. Resident skin bacteria preserve health by establishing a pH barrier that reduces pathogen colonization of the teat canal [18]. Systemic mapping of these compartmentalized communities reveals highly coordinated networks of microbes across the host body. As illustrated in Figure 1, the anatomical distribution of the bovine microbiome exhibits distinct taxonomic dominance tailored to the specific physiological demands of the rumen, lower gastrointestinal tract, mammary tissue, and skin barrier.

The four prominent phyla shared in bovine microbiome niches are *Firmicutes*, *Bacteroidetes*, *Proteobacteria*, and *Actinobacteria* [19]. *Ruminococcus* and *Butyrivibrio* are involved in fiber fermentation in the rumen, *Prevotella* is involved in starch and protein degradation, and enteric CH4 production is dominated by methanogenic archaea of the genus *Methanobrevibacter*. Lower gastrointestinal bacterial communities, such as *Bifidobacterium*, *Faecalibacterium*, and *Lactobacillus*, have been shown to support gut barrier integrity and mucosal function [8,20]. VFAs produced during rumen fermentation of cellulose and hemicellulose are used for milk fat synthesis and gluconeogenesis, and approximately 70% of the net energy expenditure of the cow is used for these two processes [21]. At the genus level, the methanogenic archaea are a diverse and fast-growing group of microorganisms, and taxonomic resolution beyond the genus level is becoming recognized as vital for targeted intervention design. In one example of a rumen archaeal community, *Methanobrevibacter* was the most abundant taxon with 63.2% of the sequences detected, followed by the *Methanomicrobia* with 9.8%, *Methanosphaera* with 7.7%, and the uncultured *Thermoplasmatales* cluster with 7.4% [22]. These values reflect the composition of the cited dataset and are expected to vary by breed, diet, and geographical location rather than representing a universal archaeal profile. Within *Methanobrevibacter*, there are two phylogenetically distinct clades: the SGMT clade (*M. smithii*, *M. gottschalkii*, *M. millerae*, *M. thaurei*) and the RO clade (*M. ruminantium*, *M. olleyae*) [22]. The animals in the SGMT clade (especially *M. gottschalkii*) consistently have significantly higher individual methane emissions than those in the RO clade, and a substantial amount of inter-animal variation in methane emissions is not explained by total archaeal abundance alone [23]. Methane is primarily synthesized through the hydrogenotrophic pathway, in which H_2_ and CO_2_ generated during bacterial, fungal, and protozoal fermentation are reduced to CH_4_, and formate accounts for up to 18% of the enteric methane produced [24]. This heterogeneity at the clade level is directly relevant to the precision-targeted CRISPR and synthetic biology strategies described in Section 5.4, because targeted removal of the entire group of archaea would affect fermentation, whereas clade specificity would not affect overall rumen function.

In addition to bacteria and archaea, the rumen hosts a eukaryotic microbial community and a complex virome, both of which have largely been overlooked in research on rumen microbiome engineering. *Neocallimastigomycota* (the anaerobic fungi) make up about 8–10% of the total microbial biomass in the rumen and include the genera *Neocallimastix*, *Piromyces* and *Orpinomyces* [25]. They exhibit rhizodial growth, can enter the structural matrix of plant cell walls, and use cellulase and hemicellulase enzyme complexes that synergize with bacterial communities to enhance fiber degradation, particularly the degradation of lignocellulosic components that can only be hydrolyzed by fibrolytic bacteria. Direct predation of other protozoa and bacteria by ciliate protozoa, dominated by the genera *Entodinium* and *Epidinium*, regulates microbial community composition, thereby controlling biomass turnover, nitrogen cycling, and hydrogen flux in the rumen. Ciliate protozoa produce a substantial amount of H_2_ and are able to transfer this hydrogen to methanogenic archaea through interspecies hydrogen transfer; methanogenesis consistently decreased by 8–20% in controlled studies after protozoa were removed, confirming that these protozoa act as indirect but quantifiably important drivers of methanogenesis [26]. Rumen bacteriophages also represent another level of regulation: a characterized core rumen virome infects at least 1051 bacterial genera, 25 archaeal genera, and 13 protozoan genera, drives a lytic cycle in the dominant rumen taxa, and enables horizontal gene transfer in the rumen through transduction. Compensatory shifts in the fungal, protozoal, or viral fractions may also occur in response to interventions targeting only the bacterial fraction, an important consideration when designing and evaluating microbiome engineering interventions [27].

Amino acids are supplemented by microbial protein, which is synthesized in the rumen. Methane production is one of the measurable energy losses, with 2–12% of gross energy intake being lost through eructation. The redirection of hydrogen flux toward propionate production also enhances feed efficiency and reduces greenhouse gas (GHG) emissions [28]. Microbial community assembly begins at birth through exposure to the dam, colostrum, and farm environment. Early microbial communities impact immune system development and function in adulthood [29]. Dietary regime is the most powerful continuous factor affecting community composition. High forage rations select for fibrolytic taxa, whereas high-concentrate rations cause a shift to *Prevotella* and lactate-producing bacteria and predispose animals to subacute ruminal acidosis (SARA) [30]. Subacute ruminal acidosis, defined as sustained rumen pH depression below 5.6 for more than three hours per day, is among the most prevalent and economically consequential dysbiosis-driven conditions in high-producing dairy cows, with reported herd-level prevalence ranging from 11% to 26% during early and peak lactation [31,32]. Higher dietary concentrate levels also negatively affect productivity and community stability by altering fermentation stoichiometry and nitrogen utilization efficiency [33]. Among the most disruptive documented perturbations is antibiotic administration, and evidence suggests that microbiome recovery may take many weeks after a single course of antibiotics [15].

An important advancement in the development of rumen microbial ecology has been the discovery of rumen enterotypes, recurring states of rumen microbial composition that group dairy cattle into biologically meaningful categories based on farm, geographic location, or management system. In dairy cows, there are two well-described enterotypes in the rumen: Rumen Prevot (R-Prevot) and Rumen Butyrivibrio (R-Butyri). R-Prevot enterotype cows have a more cohesive, more deterministically structured rumen community and produce a much larger amount of milk during lactation than R-Butyri cows [34]. Multi-omics analyses have also revealed that the rumen enterotype enriched with *Ruminococcus* species produced higher rumen propionate levels resulting in higher hepatic gluconeogenesis rates and increased amino acid availability for casein production, thus creating a mechanistic link between enterotype composition and milk protein yield [35].

## 4. Microbiome Dysbiosis and Disease Susceptibility

**Figure 2:**
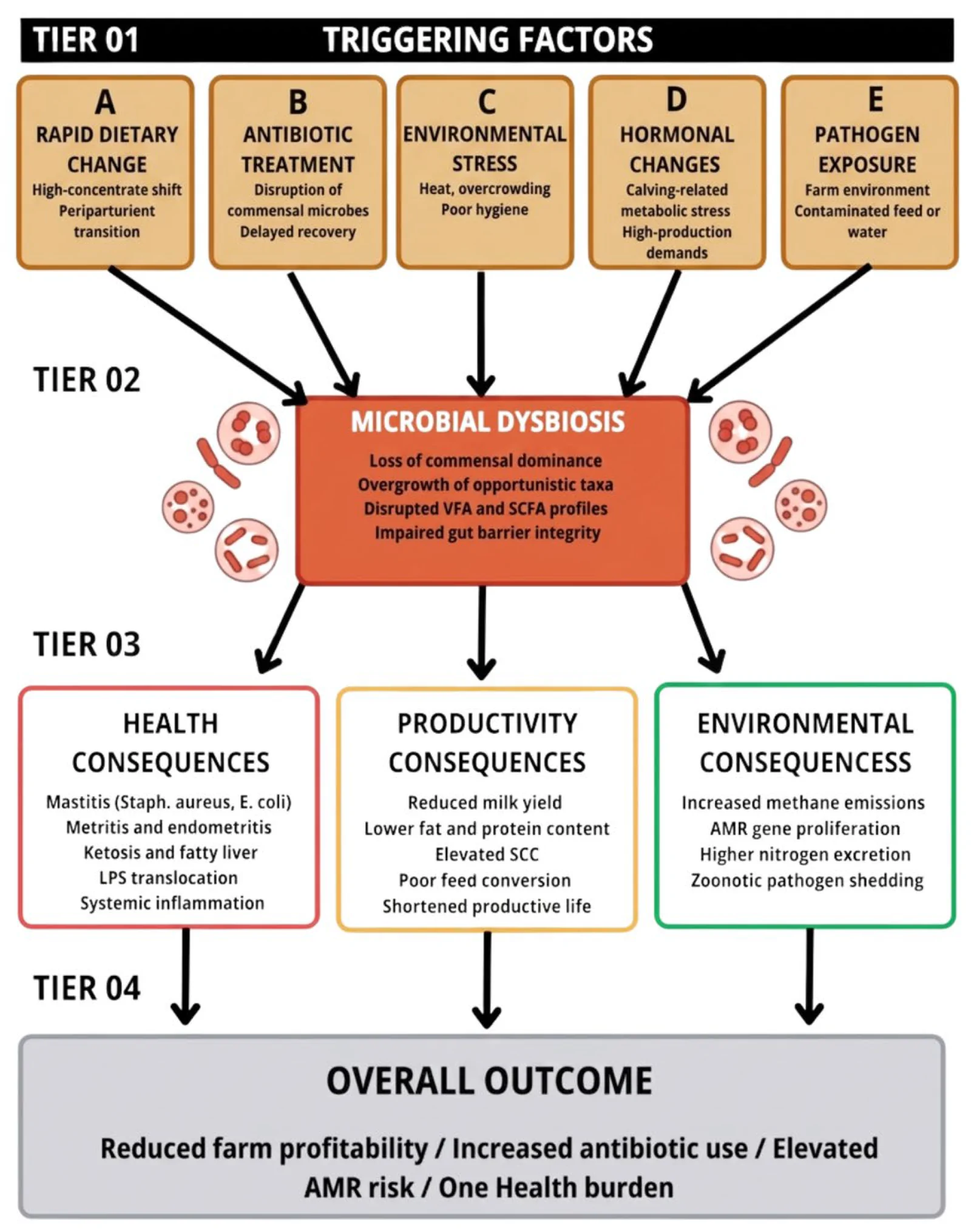
Dysbiosis cascade in dairy cows with disruption of microbial community balance as a result of dietary changes, antibiotic administration, heat stress and periparturient changes resulting in impaired VFA production, increased gut permeability, translocation of LPS and systemic inflammation. Downstream effects are a 10–25% decrease in milk yield, higher SCC, higher methane production (2–12% of gross energy) and buildup of antimicrobial resistance genes. Created in BioRender. Ashfaq, S. (2026); https://BioRender.com/kx4jp5e.

Dysbiosis is a disruption of the structure and/or function of microbial communities in terms of the number or type of microorganisms [36]. Commensal microorganisms maintain efficient digestive function, prevent the growth of facultative microorganisms, and regulate innate immune tone under conditions of microbial dysbiosis. When this balance is disrupted, the risk of metabolic diseases, reduced productivity, and infectious diseases increases significantly [37]. A transition from a homeostatic state to clinical pathology follows a distinct hierarchical path triggered by compounding operational stressors. This multi-tiered degradation pathway is illustrated in Figure 2, outlining how initial triggering factors cascade downward through microbial dysbiosis into systemic physiological damage and overall farm economic losses.

The periparturient period is the most critical period of dysbiosis in dairy animals [38]. Rapid transitions in dry matter intake alter enteral fermentation stoichiometry, increase *Prevotella* and lactate-producing populations, and enhance enteral permeability, thereby increasing LPS translocation into portal circulation [39]. Further factors that affect gut barrier function and increase pathogen shedding include heat stress, crowded housing conditions, and inadequate hygiene [40]. When therapeutic antibiotics are clinically indicated, the commensal population is depleted along with the targeted pathogens, providing an open niche for opportunistic taxa [41]. This depletion results in low-grade chronic inflammation, defective nutrient uptake, and reduced innate immune competence, with long-term consequences [42]. Microbial etiology is not limited to pathogen challenge, and mastitis is the most expensive infectious disease in dairy cattle. The restriction of udder microbiome diversity, accompanied by the emergence of *Staphylococcus aureus* or *Escherichia coli*, disrupts local immune homeostasis and increases susceptibility to mastitis [43]. The limitations of conventional antimicrobial therapy, including reduced antimicrobial penetration, biofilm-associated tolerance, and intracellular persistence of pathogens, have further encouraged the development of microbiome-based interventions as complementary preventive approaches [44]. The newly described gut microbiome-mammary axis has shifted the research focus towards systemic microbiome modulation as a preventive approach to mastitis [45].

Similar connections have been found between dysbiosis of the reproductive tract and a higher prevalence of metritis and endometritis, which are linked to lower conception rates [16]. Gut dysbiosis leads to LPS translocation into the portal vein and subsequently to low-level chronic inflammation, insulin resistance, and hepatic lipid accumulation [46]. Multi-omics studies have shown that dairy cows with fluctuating rumen microbial communities around the peri-calving period have lower dry matter intake (DMI), longer negative energy balance (NEG), decreased milk yield, and increased SCC compared to dairy cows with stable rumen microbial communities [10].

## 5. Microbiome Engineering Strategies

Bovine microbiome engineering covers a wide spectrum of strategies, from nutritional supplements to direct genomic modification of the species [47]. All these approaches aim to intentionally modify the composition or function of the microbial community to yield measurable benefits to the host [17]. Table 2 provides a comparative overview of the five principal strategies, their documented quantitative benefits, key limitations, and current TRL classifications. The quantitative ranges reported in Table 2 should be understood as a range of findings reported across individual studies, not as a range of effect sizes that can be expected in a typical dairy herd, due to differences in experimental design, breed, diet and production system.

A clear trend toward translational maturity is evident in the TRL classification. Probiotics are at the top of the TRL, with decades of commercial applications and a large body of evidence from controlled trials. Prebiotics fall within TRL 6–7, with most limitations stemming from inconsistent dose–response relationships. FMT has a TRL of 5–6 with good efficacy results in laboratory studies but has yet to be standardized for use in cattle. Metagenomic tools fall at TRL 4–5, as the correlative biomarkers they produce have not yet been demonstrated as decision-support tools in routine farm practice. CRISPR-based techniques are still at TRL 2–3, and important regulatory and biosafety issues need to be resolved prior to a scientifically and ethically acceptable field trial.

### 5.1. Probiotics and Prebiotics

Probiotics are defined as living microorganisms that, when consumed in sufficient amounts, provide a beneficial effect on the host, and prebiotics are substrates that are nondigestible by the host and promote the growth of probiotics [52]. The genera studied in dairy cattle to the greatest extent are *Lactobacillus*, *Enterococcus*, and *Saccharomyces*. These genera also enhance fiber degradation, maintain a stable rumen pH, and boost VFA production [48]. These include competitive exclusion of pathogens, production of bacteriocins, and modulation of Toll-like receptor signaling in the intestinal epithelium [49]. Dietary prebiotics, such as mannan-oligosaccharides, selectively promote commensal fiber-fermenting bacteria and reduce mucosal inflammation [64]. One delivery challenge that has prevented efficacy from being observed in the field is the viability of the probiotic strain in the gastrointestinal tract; however, this problem has been significantly addressed by encapsulating the strains [65].

A meta-analysis of controlled studies showed that multi-strain probiotic supplementation resulted in an average increase of 0.9 kg/d in milk yield. Studies showed significant heterogeneity, which was attributable to the animals’ dietary backgrounds, animal strains, and lactation stage [50]. These differences suggest that efficacy depends on how well the strains and dosing regimens are selected, rather than on the overall type of intervention. It is unlikely that any generic or off-the-shelf probiotic formulations can replicate the results achieved with probiotics specifically chosen to fit the host and farm conditions [66].

### 5.2. Fecal Microbiota Transplantation

FMT involves the transplantation of a whole microbial community from a healthy donor to a recipient animal to re-establish the recipient’s disrupted microbial community [54]. Two applications have been studied most extensively. One is early life neonatal calf transplantation to accelerate microbiome maturation. The second is restoration in adult lactating cows after antibiotic treatment or ruminal acidosis (RA) [54]. FMT in neonatal calves has been shown to promote microbiome and immune system maturation and reduce gastrointestinal diseases in early life [67]. After a rumen disturbance, FMT partially restores the ability of adult cows to ferment feed efficiently and utilize nitrogen; the magnitude varies with donor quality. The use of convenience donors has been shown to adversely affect post-transplant outcomes, an effect that can be avoided by selecting donors based on proven metabolic performance [56].

However, there are several practical limitations to the extensive use of FMT. The source of the transplanted community is a whole microbiome donor sample; therefore, its composition is never precisely defined. The requirements for biosafety screening contribute to the cost and complexity of the procedure, and there are currently no standardized commercial protocols for bovine FMT in most regulatory jurisdictions [68]. The focus of research has now shifted to well-characterized synthetic microbial consortia that can mimic the functional outcomes of whole-community FMT while further minimizing the risk of transmitting unwanted pathogens [69].

### 5.3. Genomic and Metagenomic Tools

The microbial community composition, function, and metabolism in dairy cattle have been characterized in detail using high-throughput sequencing [57]. Tools available include 16S rRNA amplicon sequencing (an affordable approach to obtain taxonomic profiles), shotgun metagenomics (which provides information on the functional gene repertoire with higher resolution), and meta-transcriptomics (which captures gene expression at a specific time point). Amplicon data can detect changes in community composition, metagenomics can detect resistance gene carriage and fermentation potential, and meta-transcriptomics can detect active metabolic changes in response to changes in the animal’s diet or environment.

A specific microbial profile has been found associated with high feed efficiency (R^2^ = 0.6–0.8) and with reduced methane production (prediction error of about 10%) and with susceptibility to subacute ruminal acidosis (sensitivity of 70–85%) [59]. These predictive models have been created, however, largely using single study populations and are typically only evaluated in the original data sets. External validation across independent herds has been reported rarely, reducing confidence in their robustness and wider applicability. Machine learning models incorporating microbiome variables, animal genome data, and production records have been used to predict individual animal responses to dietary interventions and the addition of probiotics to feed [70]. The coefficient of variation (CV) from the predictive model indicates a biological variation of 20–40% that cannot be fully explained by the existing analytical methods. Correlational, rather than interventional, study designs are more common, limiting the ability to establish causal associations between microbial traits and production outcomes, except in well-powered interventional studies [70].

### 5.4. CRISPR and Synthetic Biology

CRISPR-based genome editing enables the modification of microbial functionality at the gene level [38], whereas synthetic biology enables modification at the community functional level [71]. These technologies have been used to research the inactivation of methanogenesis genes in rumen archaea, improve cellulose fermentation efficiency in rumen bacteria, and improve thermal and oxidative stress resilience in probiotic strains [62]. Engineered rumen bacteria have been shown to achieve 15–30% greater cellulose degradation and return more energy under controlled laboratory conditions [63]. Synthetic biology takes these abilities a step further by enabling the design of entire microbial communities that work together to perform related functions, such as pathogen suppression, nitrogen recycling optimization, and ruminal pH stabilization [60].

However, the commercialization of these technologies is still distant. Most jurisdictions lack or have not fully developed regulatory systems to address Living modified organisms (LMOs) in food-producing animals. A critical biosafety concern regarding the open rumen environment is the high probability of horizontal gene transfer (HGT). Engineered genetic elements can potentially spread to non-target wild-type microbial species via plasmid transfer, leading to unpredictable ecological outcomes. Therefore, it is important that the persistence of the engineered strains is monitored over the long term in manure, soil, and water resources before a field trial is conducted. There needs to be transparent, research-based biosafety programming and broad consultation with stakeholders long before any evaluation at the field scale [60,72].

## 6. Impact on Dairy Productivity and Health

In controlled trials, microbiome-based interventions have been associated with improvements in milk volume, composition, SCC, feed conversion efficiency, and antibiotic use [33]. However, efficacy in commercial farm trials is lower and less predictable than that in controlled research trials and is affected by variability in feeding systems, housing, and farm management [73]. Controlled trials conducted in different environments have reported a mean increase in milk yield of 0.9 kg/day (range, 0.5–1.5 kg/day) and a mean increase in milk fat of 0.12% [51,74]. Such improvements appear to be associated with changes in fermentation that increase propionate and butyrate production, both of which are beneficial for milk yield and quality [46,75]. The SCC is decreased by 20–40% due to improved udder and gastrointestinal microbial balance, reflecting a reduced burden of subclinical mastitis. Feed losses can be reduced by 5–10%, and methane emissions per kg of milk production by 10–20%, by optimizing rumen microbes for efficient fermentation, providing both economic and environmental benefits [76].

There is sufficient literature demonstrating health-related effects. In several controlled trials, the incidence of mastitis was reduced by 30–50%, and antibiotic use by 40–60% with multi-strain probiotics and symbiotics [40,77]. The responsiveness of neonatal calves to early microbiome modulation is especially evident, with weaning mortality reduced by 5–10% and increased first-lactation yield attributable to early-life interventions [78,79]. Evidence supporting a national AMR reduction strategy has been presented, showing improved productive performance with microbiome-based approaches rather than antimicrobial growth promoters based on long-term farm-level investigations [80].

Economic analyses in various countries have shown that net financial returns per cow per year, with increased milk production of USD 50 to USD 150, decreased treatment costs of USD 20 to USD 50, and reduced involuntary culling rates. Higher microbial fermentation efficiency is estimated to reduce GHG emissions per kg of milk by 10–15%. The remaining diversity in the results across trials indicates that one-size-fits-all approaches to dose rates are inadequate for field performance. The main factors that ensure reproducible outcomes are strain selection, timing of administration, and host-microbiome compatibility [81].

## 7. Integration with Precision Livestock Farming

**Figure 3:**
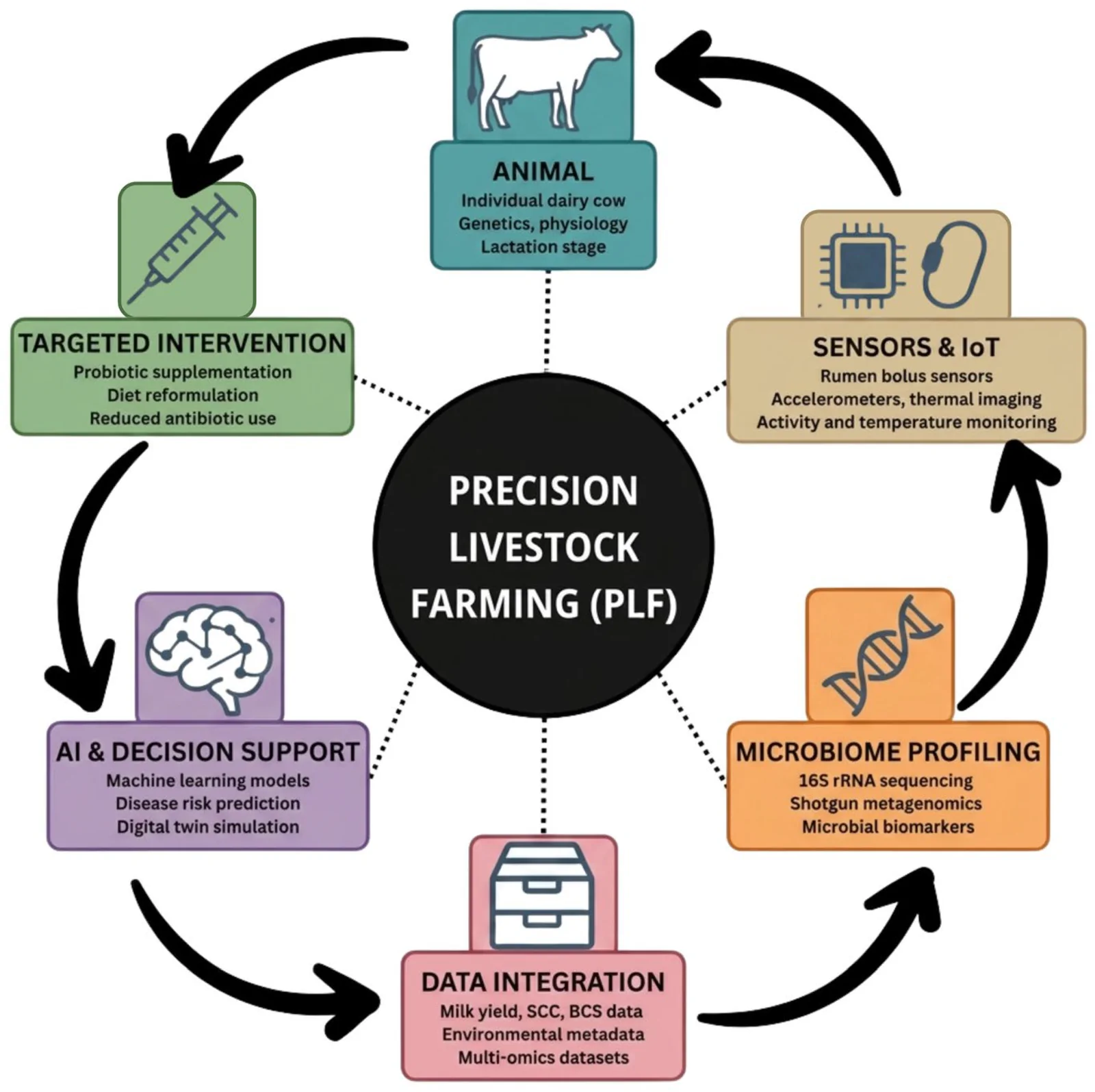
Precision livestock farming feedback loop connecting real-time sensor data and microbiomes to AI-driven decision systems for targeted nutritional and probiotic individual animal interventions. Created in BioRender. Ashfaq, S. (2026); https://BioRender.com/sk7dmuk.

Precision livestock farming (PLF) is a technology-embedded management strategy that includes real-time monitoring sensors, microbiome profiling, and machine learning analytics, enabling evidence-based decisions at the animal level [82]. PLF represents a shift away from a uniform herd-level approach to interventions informed by the physiology and microbiology of individual animals at specific time points. To bridge this gap, modern management requires an automated loop capable of pairing real-time data capture with biological tracking systems. This integrated management paradigm is operationalized in the feedback loop shown in Figure 3, demonstrating the continuous flow of information from automated sensors to AI-driven diagnostic interfaces that ultimately execute targeted animal-specific probiotic or nutritional adjustments.

Data from non-invasive rumen gas monitors, body temperature, and behavioral monitors can be correlated with microbial activity data to enable the early detection of metabolic instability before the appearance of clinical signs [83]. A more complete definition of the internal physiological state can be achieved by integrating accelerometers, rumen bolus pH monitors, and thermal imaging cameras into a single sensor platform. If continuous sensor data are supplemented with time-series microbial sequences, it becomes possible to detect dysbiosis in individual animals and trigger corrective interventions before the clinical onset of the disease [84].

In some cases, machine learning models that combine microbiome, genomic, and production data have proven to be clinically relevant and accurate. The reported sensitivities are 75–85% for subacute ruminal acidosis, 70–80% for mastitis and 80–85% for negative energy balance, though external validation across independent farms was not consistently reported for these models either [85]. Such predictive outputs can help guide the development of personalized nutrition and probiotic treatment plans for each animal, rather than herd-wide blanket approaches. A computational model of an individual animal, built from microbiome profiles, physiological measurements, and environmental data, can also simulate animal responses to dietary changes or the addition of a dietary supplement (probiotic) without first implementing the change in the real world [86]. It is still in a very early stage of development and, for now, has the handicap of requiring a large number of computers and lacks validation data from commercial farms over the longer term. In the future, Internet of things (IoT) technology in delivery systems is expected to enable automated real-time adjustments to individual animal nutrition and probiotic dosages [86].

## 8. One Health Implications

The One Health concept recognizes the interconnection between animal, human, and environmental health. Engineering the Microbiome in Dairy has direct implications in all three domains [87]. It has been shown that manipulating rumen microbial function can reduce enteric CH4 emissions by 10–20% without negatively affecting feed efficiency or milk yield. Improved efficiency of rumen N utilization decreases the nitrogen load in the air, water, and soil systems by reducing N volatilization from manure by 15–25%. However, some interventions to lower CH_4_ production may increase N_2_O emissions from manure, creating environmental trade-offs that must be quantified before a particular intervention can be advocated as an overall environmental improvement [88].

There is a well-established link between microbiome engineering and AMR mitigation. The resistance genes found in rumen and gut microbes are spread to the broader environment via manure, runoff water, and soil transfers. The use of routine prophylactic antibiotics leads to the accumulation of resistance genes, which can be effectively reduced through nutrition- and microbiome-based interventions. In long-term controlled studies, a 40–60% reduction in antibiotic use did not result in decreased productive performance [89].

The rumen resistome, the full set of antimicrobial resistance genes (ARGs) present in the rumen microbiome, has been systematically characterized, adding an additional layer to the One Health aspects of bovine microbiome management. The rumen content of dairy cattle was subjected to metagenomic surveys that have identified more than 900 ARGs from at least 26 classes of antimicrobials, being tetracycline resistance genes the most common, followed by macrolide resistance genes such as macB, which were found at around 0.05% relative abundance in all herds examined in a representative multi-herd survey [90]. Importantly, high-concentrate diets have also been associated with increased diversity and abundance of antimicrobial resistance genes, even in the absence of antibiotic administration, suggesting that dietary management may contribute to ARG accumulation within the rumen microbiota. This relationship is currently supported by observational evidence and should therefore be interpreted as an association rather than a demonstrated causal mechanism [91]. The rumen microbiome is a source of ARGs that escape into the external environment in three main ways: by direct excretion in feces, by land use of manure, and by contamination of milk during milking due to disruption of the gut-mammary microbiome. The host genetic contribution to ARG carriage has also been quantified, and heritable components of the rumen microbiome have been found to be associated with ARG carriage across herds, providing a theoretical framework for targeting low-ARG rumen microbiome traits as an additional One Health intervention alongside rumen microbiome engineering [90,92].

Microbiome stability is also important for food safety. Farms with stable gastrointestinal and rumen microbial communities have lower on-farm colonization by foodborne pathogens, such as *Escherichia coli* O157:H7 and *Salmonella enterica* [93]. Continuous monitoring of the microbiome could be implemented as a standard part of dairy operations and serve as an early warning system for emerging pathogen threats and resistance mechanisms, thereby improving the coordination between veterinary and public health monitoring systems [94].

The potential impact of engineering rumen microorganisms to reduce enteric methane emissions on the Greenhouse Gas Trajectory (GHGT) of Animal Respiratory Diseases (ARDs) remains an open question. The possibility of such an interaction has not been systematically investigated and should be the focus of research before considering sophisticated genetic engineering for commercial livestock use [94].

## 9. Challenges and Limitations

One of the most longstanding constraints in the field is the gap between efficacy in controlled research environments and the results on commercial farms [95]. Table 3 summarizes the main groups of barriers to field translation and suggests evidence-based solutions for each group.

The most basic challenge to consistent field performance is the biological variability. Many factors affect microbial community composition, including host genetics, nutrition, housing environment, and management practices, which can lead to large variations among individuals, between herds, and between farm systems. The rumen microbiome is highly ecologically resilient and usually regenerates to its ‘pre-intervention’ normal state within days to weeks of any event. Therefore, continuous administration or encapsulation systems are necessary to prolong colonization if changes in the community are to be achieved permanently. These demands are among the reasons for the discrepancies often observed between test results and field conditions [97].

The second major barrier is the technical limitations of data generation and analysis. In addition, there has been no standardization across studies for sample collection, sequencing depth, or bioinformatic processing, which makes it difficult to compare results across studies and create reliable reference data. Most studies are correlational; therefore, there is limited evidence of causality regarding specific microbial attributes and health and productivity outcomes. To fill this gap, large-scale interventional studies with sufficient power are necessary, a requirement that has been logistically difficult for individual research groups [99].

Comparatively less attention has been paid to host genome architecture as a source of biological variability in the literature on microbiome engineering. A proportion of the inter-animal variation in rumen community composition is heritable, as suggested by genome-wide association studies and multi-farm, multi-omics analyses, with heritability estimates (*h*^2^) of individual taxa ranging from 0.10 to 0.35. A landmark multi-farm study revealed that there is a shared, core heritable rumen microbiome present in a variety of farm environments, with heritable loci of the microbiome types reproducibly detected across distinct rumen populations, and that the pooled microbiome composition of an individual animal could be partly predicted from a genomic profile alone [105]. The two key translational implications of this host-microbiome genetic co-regulation are provided below. Firstly, it offers a powerful explanation for why different probiotic products given to similar herds under identical dietary conditions yield different results across genetically distinct herds: the recipient’s genetic makeup will limit which modifications can be ecologically stable over time. Second, it also offers an alternative approach using genomic selection in which breeding indices that include low levels of methanogens, high diversity in fibrolytic bacteria, or high abundance of *Succinovibrionaceae* as heritable traits within the microbiome could facilitate ongoing herd improvement without continual use of intervention products, and should be explored in conjunction with direct microbiome engineering [105,106].

The third class of constraints is regulatory, economic, and biosafety barriers. There is no clear regulatory definition of engineered probiotics or synthetic microbial consortia in most countries, which hinders product approvals and commercial investments [60]. Smallholder and low-margin producers face further barriers to the uptake of metagenomic analysis because of the cost (USD 50–200/sample) and the lack of well-defined return profiles. The biosafety concerns associated with synthetic biology applications in livestock systems are well-founded and cannot be dismissed on the basis of anticipated production benefits alone. Prior to the field use of engineered microorganisms, an ecological impact assessment is required [67,101].

## 10. Future Directions and Research Priorities

Three priority areas across the dairy microbiome that will be key to advancing the field of microbiome engineering over the next decade are personalization of interventions, adaptation to climate change, and the establishment of a common data platform. Advances in one area depend on simultaneous advances in other areas.

Personalized microbiome management is the most direct solution to biological variability. Currently, the same formulations are used across the entire herd, even though there are significant differences in the individual baseline microbial status and likely in the treatment response [107]. To address this, a design with randomization of animals according to their original rumen enterotype and specific evaluation of probiotic formulations by enterotype through subsequent assessment of milk production, methane production, and SCC at specific time points should be considered. Certain microbial markers (such as “*Prevotella* over *Ruminococcus*”) have yet to be validated as reliable predictors of individual animal response to microbiome-based interventions in dairy cattle production systems. To make this level of precision operationally feasible, affordable on-farm sequencing instruments capable of generating microbiome profiles within 24 h are needed [108].

Adaptation to climate change is an emerging research priority. Rumen stability and fermentation efficiency are already influenced by heat stress, decreasing water availability, and increased fluctuations in feed quality, all of which are expected to increase in the main dairy-producing areas under future climate scenarios [109].

Recent metagenomic investigations have begun to uncover the basis of thermal resilience in dairy cows and to offer empirical support for developing microbiome-based climate adaptation strategies. The heat-resistant cows exhibited more integrated rumen microbiome networks than the heat-sensitive animals, and the heat-resistant rumen had a higher abundance of *Ruminococcus flavefaciens* and *Succiniclasticum* and was enriched in genes involved in the pentose phosphate pathway (a metabolic adaptation that improves coping with oxidative stress during thermal challenge) [110]. By contrast, heat-sensitive cows experience greater metabolic stress in the rumen, as indicated by higher activity of the TCA cycle and pyruvate metabolism in the rumen microbiome under the same thermal environment [110]. With increasing heat stress, the abundance of the fibrolytic taxa decreases, and that of the opportunistic taxa increases, and this community change is associated with decreased ability to maintain volatile fatty acid homeostasis, lower dry matter intake, and lower milk yield. In addition, breed-specific microbial responses to heat stress have been reported, and Holstein cows were more adaptable in their microbiome responses to thermal stressors than Jersey cows, which may be partly due to differences in their baseline bacterial and fungal community profiles. These observations need to be translated into actionable climate adaptation strategies, which in turn requires three research priorities: the isolation and characterization of thermostable fibrolytic variants of the microbiome, notably heat-tolerant variants of *Ruminococcus flavefaciens* and *Fibrobacter succinogenes*; climate adaptation of candidate probiotic strains using the CRISPR approach; and design of controlled field trials investigating the effectiveness of microbiome supplementation programs for heat-stressed dairy herds in subtropical and tropical production systems, which represent more than 60% of the world’s dairy cattle population. The isolation of heat-tolerant fibrolytic bacterial strains, the engineering of selected strains to improve thermal resistance using CRISPR tools, and the design of field trials to test conventional and climate-adapted bacterial probiotic supplementation in heat-stressed dairy herds are prioritized research activities [111].

Data infrastructure development is the third priority and is cross-cutting to other priorities. Research on the cattle microbiome is burgeoning; however, there are currently no interoperable databases that link microbial data to animal performance, clinical health events, and farm management data. Meta-analyses lack reliability if they do not include these databases; the validation of a biomarker across different populations is impossible, and machine learning models cannot be generalized beyond the herds on which they were trained [98]. Interdisciplinary collaboration among microbiologists, veterinarians, data scientists, and policymakers is needed to establish FAIR (Findable, Accessible, Interoperable, Reusable) systems for data collection, access governance, and biosafety reporting, and this collaboration must be ongoing [112].

## 11. Conclusions

Microbiome engineering represents a significant paradigm shift in modern dairy production, providing a viable path to improve livestock productivity, enhance disease resistance, and alleviate the environmental impacts. While the traditional use of probiotics and prebiotics reveals high translational maturity (TRL 7–8), cutting-edge strategies, such as synthetic consortia and CRISPR-based modifications, remain constrained at earlier developmental stages (TRL 2–3). A key insight of this synthesis is that field-level efficacy is primarily dictated by host-microbiome compatibility and baseline enterotypes rather than generic product design. To transition from uniform herd-level administration to reproducible precision interventions, the field must integrate longitudinal multi-omics profiling with real-time sensor analytics within precision livestock farming systems. Overcoming these persistent bottlenecks will require global bioinformatic standardization, open-access livestock data repositories, and updated regulatory frameworks for engineered biologics. Ultimately, addressing these structural and organizational hurdles through interdisciplinary collaboration is critical to unlocking the full potential of precision microbiome management within the global One Health framework.

## Figures and Tables

**Table 1:** Anatomical localization of the microbiome of dairy cows: taxonomic dominance, functional importance, and quantitative health significance.

Body Site	Dominant Taxa	Primary Function	Quantitative Health Relevance (Range)	References
Rumen	*Ruminococcus*, *Prevotella*, *Butyrivibrio*, methanogenic archaea	Cellulose fermentation to VFAs, microbial protein synthesis	VFA yield: 60–80% of energy [7]; methane loss: 2–12% of gross energy [8,9]	[7,8,9]
Gastrointestinal tract	*Bifidobacterium*, *Faecalibacterium*, *Lactobacillus*	SCFA signaling, mucosal immunity, pathogen exclusion	SCFA: 50–120 mmol/kg digesta [10]; LPS translocation risk triples in dysbiosis [11]	[10,11]
Udder (mammary)	*Staphylococcus*, *Corynebacterium*, *Lactococcus* (commensals)	Local immune priming, competitive exclusion of mastitis pathogens	Healthy SCC below 100,000 cells/mL [12]; dysbiosis linked to SCC above 200,000 cells/mL [13]	[12,13]
Skin	*Pseudomonas*, *Acinetobacter*, commensal *Staphylococcus*	Antimicrobial peptide production, maintenance of skin pH barrier	Disruption increases teat canal colonization risk two- to three-fold [14,15]	[14,15]
Reproductive tract	*Prevotella*, opportunistic *Fusobacterium*, *Escherichia*	Immune modulation of endometrium, regulation of inflammatory cytokines	Dysbiosis correlates with 15–25% lower conception rates [16,17]	[16,17]

**Table 2:** Comparative overview of microbiome engineering strategies in dairy cattle, ranked by Technology Readiness Level (TRL 1–9).

Strategies	Mechanism of Action	Quantitative Benefits (Range)	Key Limitations	TRL	References
Probiotics	Competitive exclusion, antimicrobial compound production, immune modulation, ruminal pH stabilization	Milk yield: +0.5–1.5 kg/d; SCC reduction: 20–40%; feed efficiency: +5–10%	Strain-specific efficacy; viability loss in gastrointestinal transit; inconsistent outcomes across farms	7–8 (field ready)	[48,49,50,51]
Prebiotics	Selective stimulation of beneficial microbes, improved gut barrier function, SCFA upregulation	Gut diversity: +10–30%; milk fat: +0.1–0.3%; inflammation markers: −15–25%	Limited species-specificity; variable dose–response; cost constraints	6–7 (ready with refinement)	[52,53]
Fecal microbiota transplantation (FMT)	Community-level transfer from healthy donor, restoration of taxonomic and functional diversity	Calf gut maturation accelerated by 2–3 weeks; rumen fermentation recovery 40–60% of baseline within 7 days	Biosafety concerns; donor variability; absence of standardized protocols; pathogen transfer risk	5–6 (promising, protocol-dependent)	[54,55,56]
Genomic and metagenomic tools	16S rRNA sequencing, shotgun metagenomics, metatranscriptomics, machine learning integration	Feed efficiency prediction R^2^ 0.6–0.8; methane prediction error ±10%; disease sensitivity 70–85%	High cost; absence of standardized bioinformatic pipelines; predominantly correlative evidence	4–5 (research tool)	[57,58,59]
CRISPR and synthetic biology	Targeted gene editing of rumen microbes, biosensor-based circuits, synthetic consortia design	Methane reduction: 10–20% in trials; fiber degradation: +15–30%; pathogen suppression: 2–5× greater than controls	Regulatory ambiguity; biosafety and ecological risks; horizontal gene transfer concerns	2–3 (pre-commercial)	[60,61,62,63]

**Table 3:** Microbiome engineering in dairy cattle: critical challenges, implementation impacts, and proposed solutions.

Challenge Category	Specific Barrier	Impact on Implementation	Proposed Solution	References
Biological variability	Host genetics, diet, environment, and farm management produce highly heterogeneous microbial communities	Inconsistent intervention outcomes, poor reproducibility across herds and regions	Develop host genotype-stratified interventions [96]; conduct multi-farm longitudinal studies [97]	[96,97]
Microbiome resilience	Rumen microbiome frequently reverts to baseline following manipulation; limited colonization persistence	Short-lived benefits; requirement for repeated or continuous dosing	Engineer persistent microbial strains [98]; develop encapsulation technologies for sustained delivery [65]	[65,98]
Data standardization	Absence of harmonized protocols for sampling, sequencing depth, and bioinformatic analysis	Cross-study incompatibility; delays in biomarker validation and meta-analysis	Adopt international sequencing standards [99]; establish open-access livestock microbiome databases [100]	[99,100]
Regulatory barriers	Engineered probiotics and synthetic consortia lack a clear regulatory classification in most jurisdictions	Delayed product approvals; reduced commercial investment	Establish dedicated regulatory pathways for microbiome-based livestock products, drawing on frameworks proposed for engineered probiotics [101] and live biotherapeutic products [102]	[101,102]
Economic constraints	Unclear cost–benefit profiles; high upfront costs for omics and precision tools	Low adoption rates, particularly in low-margin or smallholder dairy systems	Develop subsidized pilot programs [73]; conduct farm-level economic assessments [103]	[73,103]
Ethical and biosafety risks	Horizontal gene transfer risk; unintended ecological consequences of releasing engineered microorganisms	Public concern, regulatory scrutiny, potential environmental contamination	Implement validated microbial containment strategies [104]; require mandatory ecological impact assessment [72]	[72,104]

## Data Availability

No new data were created or analyzed in this study. Data sharing is not applicable to this article.

## Supplementary Materials

The following supporting information can be downloaded at: https://www.mdpi.com/article/10.3390/vetsci13080766/s1. Table S1: Complete database-specific search strategies, applied filters, and records retrieved for the systematic literature search.; Figure S1: PRISMA 2020 flow diagram showing the identification, screening, eligibility assessment, and inclusion process of studies in the systematic review. Records were identified through database searches (PubMed, Scopus, Web of Science, Google Scholar, and CAB Abstracts), followed by duplicate removal, title/abstract screening, full-text assessment, exclusion with reasons, and final inclusion of studies. A total of 110 studies were included in the qualitative synthesis. Created in BioRender. Ashfaq, S. (2026) https://BioRender.com/jtkamoi.

## Abbreviations

The following abbreviations are used in this manuscript:

AI	Artificial Intelligence
AMR	Antimicrobial Resistance
ARGs	Antimicrobial Resistance Genes
CRISPR	Clustered Regularly Interspaced Short Palindromic Repeats
CV	Coefficient of Variation
DMI	Dry-Matter Intake
DNA	Deoxyribonucleic Acid
FAIR	Findable, Accessible, Interoperable, Reusable
FMT	Fecal Microbiota Transplantation
GHG	Greenhouse Gas
GHGT*	Greenhouse Gas Trajectory
HGT	Horizontal Gene Transfer
IoT	Internet of Things
LMO	Living Modified Organism
LPS	Lipopolysaccharide
NEG	Negative Energy Balance
PLF	Precision Livestock Farming
PRISMA	Preferred Reporting Items for Systematic Reviews and Meta-Analyses
RA	Ruminal Acidosis
RNA	Ribonucleic Acid
rRNA	Ribosomal Ribonucleic Acid
SARA	Subacute Ruminal Acidosis
SCC	Somatic Cell Count
SCFA	Short-Chain Fatty Acid
SCFAs	Short-Chain Fatty Acids
TRL	Technology Readiness Level
VFA	Volatile Fatty Acid
VFAs	Volatile Fatty Acids

## References

[1] Virto M., Santamarina-García G., Amores G., Hernández I. (2022). Antibiotics in Dairy Production: Where is the Problem?. Dairy.

[2] Vidovic N., Vidovic S. (2020). Antimicrobial Resistance and Food Animals: Influence of Livestock Environment on the Emergence and Dissemination of Antimicrobial Resistance. Antibiotics.

[3] FAO (2023). Pathways Towards Lower Emissions—A Global Assessment of the Greenhouse Gas Emissions and Mitigation Options from Livestock Agrifood Systems.

[4] Kaur H., Kaur G., Gupta T., Mittal D., Ali S.A. (2023). Integrating Omics Technologies for a Comprehensive Understanding of the Microbiome and Its Impact on Cattle Production. Biology.

[5] Jin Song S., Woodhams D.C., Martino C., Allaband C., Mu A., Javorschi-Miller-Montgomery S., Suchodolski J.S., Knight R. (2019). Engineering the Microbiome for Animal Health and Conservation. Exp. Biol. Med..

[6] Donadia A.B., Torres R.N., Silva H.M., Soares S.R., Hoshide A.K., Oliveira A.S. (2023). Factors Affecting Enteric Methane Emission and Predictive Models for Dairy Cows. Animals.

[7] Nagaraja T.G., Millen D.D., De Beni Arrigoni M., Lauritano Pacheco R. (2016). Microbiology of the Rumen. Rumenology.

[8] Mao S., Zhang M., Liu J., Zhu W. (2015). Characterising the Bacterial Microbiota across the Gastrointestinal Tracts of Dairy Cattle: Membership and Potential Function. Sci. Rep..

[9] Keum G.B., Pandey S., Kim E.S., Doo H., Kwak J., Ryu S., Choi Y., Kang J., Kim S., Kim H.B. (2024). Understanding the Diversity and Roles of the Ruminal Microbiome. J. Microbiol..

[10] Xue M.Y., Sun H.Z., Wu X.H., Liu J.X., Guan L.L. (2020). Multi-Omics Reveals That the Rumen Microbiome and Its Metabolome Together with the Host Metabolome Contribute to Individualized Dairy Cow Performance. Microbiome.

[11] Fuhren J., Schwalbe M., Boekhorst J., Rösch C., Schols H.A., Kleerebezem M. (2024). Prebiotic Utilisation Provides Lactiplantibacillus plantarum a Competitive Advantage in Vitro, but Is Not Reflected by an Increased Intestinal Fitness. Gut Microbes.

[12] Khasapane N.G., Khumalo Z.T.H., Kwenda S., Nkhebenyane S.J., Thekisoe O. (2023). Characterisation of Milk Microbiota from Subclinical Mastitis and Apparently Healthy Dairy Cattle in Free State Province, South Africa. Vet. Sci..

[13] Ibrahim N. (2017). Review on Mastitis and Its Economic Effect. Can. J. Sci. Res..

[14] Heizer B.T. (2020). Impact of Management Practices on Milk Quality on North Carolina Dairy Farms. Master’s Thesis.

[15] Kim S.H., Ramos S.C., Valencia R.A., Cho Y.I., Lee S.S. (2022). Heat Stress: Effects on Rumen Microbes and Host Physiology, and Strategies to Alleviate the Negative Impacts on Lactating Dairy Cows. Front. Microbiol..

[16] Adnane M., Chapwanya A. (2024). Microbial Gatekeepers of Fertility in the Female Reproductive Microbiome of Cattle. Int. J. Mol. Sci..

[17] Shaukat A., Yang C., Yang Y., Guo Y.-F., Jiang K., Guo S., Yang X., Wu H., Deng G. (2020). Ginsenoside Rb 1: A novel therapeutic agent in *Staphylococcus aureus*-induced acute lung injury with special reference to oxidative stress and apoptosis. Microb. Pathog..

[18] Derakhshani H., Plaizier J.C., De Buck J., Barkema H.W., Khafipour E. (2020). Composition and Co-Occurrence Patterns of the Microbiota of Different Niches of the Bovine Mammary Gland: Potential Associations with Mastitis Susceptibility, Udder Inflammation, and Teat-End Hyperkeratosis. Anim. Microbiome.

[19] Murga-Valderrama N.L., Segura-Portocarrero G.T., Romani-Vasquez A.C., Frias-Torres H., Flores-Durand G.J., Cornejo-Villanueva V.G., Del Solar J.C., Costa-Polveiro R., da Silva Vieira D., Bardales-Escalante W. (2023). Exploring the Microbiome of Two Uterine Sites in Cows. Sci. Rep..

[20] Chen X., Yan F., Liu T., Zhang Y., Li X., Wang M., Zhang C., Xu X., Deng L., Yao J. (2022). Ruminal Microbiota Determines the High-Fiber Utilization of Ruminants: Evidence from the Ruminal Microbiota Transplant. Microbiol. Spectr..

[21] Drehmel O.R., Brown-Brandl T.M., Judy J.V., Fernando S.C., Miller P.S., Hales K.E., Kononoff P.J. (2018). The Influence of Fat and Hemicellulose on Methane Production and Energy Utilization in Lactating Jersey Cattle. J. Dairy Sci..

[22] Danielsson R., Dicksved J., Sun L., Gonda H., Müller B., Schnürer A., Bertilsson J. (2017). Methane Production in Dairy Cows Correlates with Rumen Methanogenic and Bacterial Community Structure. Front. Microbiol..

[23] Smith P.E., Kelly A.K., Kenny D.A., Waters S.M. (2022). Differences in the composition of the rumen microbiota of finishing beef cattle divergently ranked for residual methane emissions. Front. Microbiol..

[24] de la Fuente G., Yañez-Ruiz D.R., Seradj A.R., Balcells J., Belanche A. (2019). Methanogenesis in animals with foregut and hindgut fermentation: A review. Anim. Prod. Sci..

[25] Yan M., Yu Z. (2024). Viruses contribute to microbial diversification in the rumen ecosystem and are associated with certain animal production traits. Microbiome.

[26] Belanche A., de la Fuente G., Newbold C.J. (2014). Study of methanogen communities associated with different rumen protozoal populations. FEMS Microbiol. Ecol..

[27] Yan M., Pratama A.A., Somasundaram S., Li Z., Jiang Y., Sullivan M.B., Yu Z. (2023). Interrogating the viral dark matter of the rumen ecosystem with a global virome database. Nat. Commun..

[28] Cuervo W., Gomez-Lopez C., DiLorenzo N. (2025). Methane synthesis as a source of energy loss impacting microbial protein synthesis in beef cattle: A review. Methane.

[29] Kalbermatter C., Fernandez Trigo N., Christensen S., Ganal-Vonarburg S.C. (2021). Maternal microbiota, early life colonization and breast milk drive immune development in the newborn. Front. Immunol..

[30] Souza J.M., Ribeiro P.H., Millen D.D. (2026). Shifts of rumen microbiota by feeding non-fibrous carbohydrates to improve cattle performance. Front. Microbiol..

[31] Danscher A.M., Li S., Andersen P.H., Khafipour E., Kristensen N.B., Plaizier J.C. (2015). Indicators of induced subacute ruminal acidosis (SARA) in Danish Holstein cows. Acta Vet. Scand..

[32] Plaizier J.C., Krause D.O., Gozho G.N., McBride B.W. (2008). Subacute ruminal acidosis in dairy cows: The physiological causes, incidence and consequences. Vet. J..

[33] Agle M., Hristov A.N., Zaman S., Schneider C., Ndegwa P.M., Vaddella V.K. (2010). Effect of dietary concentrate on rumen fermentation, digestibility, and nitrogen losses in dairy cows. J. Dairy Sci..

[34] Hao Y., Choi Y., Seifert J., Wang W., Wang Y.J., Cao Z., Yang H., Guan L.L., Li S. (2025). Temporal profiling of rumen and hindgut microbiota revealed enterotypes affecting the microbial interactions and assembly in the gut of dairy cows. ISME Commun..

[35] Wang D., Tang G., Wang Y., Yu J., Chen L., Chen J., Wu Y., Zhang Y., Cao Y., Yao J. (2023). Rumen bacterial cluster identification and its influence on rumen metabolites and growth performance of young goats. Anim. Nutr..

[36] Schippa S., Conte M.P. (2014). Dysbiotic events in gut microbiota: Impact on human health. Nutrients.

[37] Kivimäki M., Bartolomucci A., Kawachi I. (2023). The multiple roles of life stress in metabolic disorders. Nat. Rev. Endocrinol..

[38] Keshri A., Bashir Z., Kumari V., Prasad K., Joysowal M., Singh M., Singh D., Tarun A., Shukla S. (2021). Role of micronutrients during peri-parturient period of dairy animals—A review. Biol. Rhythm Res..

[39] Linder H.F. (2024). Further Elucidating the Effects of Ruminal and Hindgut Acidosis on the Ruminant Animal. Ph.D. Thesis.

[40] Patra A.K., Kar I. (2021). Heat stress on microbiota composition, barrier integrity, and nutrient transport in gut, production performance, and its amelioration in farm animals. J. Anim. Sci. Technol..

[41] Kim S., Covington A., Pamer E.G. (2017). The intestinal microbiota: Antibiotics, colonization resistance, and enteric pathogens. Immunol. Rev..

[42] Balamurugan B.S., Marimuthu M.M.C., Sundaram V.A., Saravanan B., Chandrababu P., Chopra H., Malik T. (2024). Micro nutrients as immunomodulators in the ageing population: A focus on inflammation and autoimmunity. Immun. Ageing.

[43] Maity S., Ambatipudi K. (2021). Mammary microbial dysbiosis leads to the zoonosis of bovine mastitis: A One-Health perspective. FEMS Microbiol. Ecol..

[44] Ban-Cucerzan A., Morar A., Tîrziu E., Bucur I.-M., Popa S.-A., Imre K. (2026). Bovine Mastitis Therapy at a Crossroads: Pharmacokinetic Barriers, Biofilms, Antimicrobial Resistance, and Emerging Solutions. Pharmaceuticals.

[45] Li Y., Wang M., Liu W., Geng M., Asiri M., Alzahrani F.M., Alzahrani K.J., Ma Q., Wang C., Khan M.Z. (2025). Targeting the gut-mammary axis for understanding mastitis pathogenesis and therapeutic strategies. Vet. Sci..

[46] Faniyi T.O., Adegbeye M.J., Elghandour M.M., Pilego A.B., Salem A.Z., Olaniyi T.A., Adediran O., Adewumi M.K. (2019). Role of diverse fermentative factors towards microbial community shift in ruminants. J. Appl. Microbiol..

[47] Perisse I.V., Fan Z., Singina G.N., White K.L., Polejaeva I.A. (2021). Improvements in gene editing technology boost its applications in livestock. Front. Genet..

[48] Retta K.S. (2016). Role of probiotics in rumen fermentation and animal performance: A review. Int. J. Livest. Prod..

[49] McCann J.C., Elolimy A.A., Loor J.J. (2017). Rumen microbiome, probiotics, and fermentation additives. Vet. Clin. North Am. Food Anim. Pract..

[50] Salah N., Legendre H., Pain P.P., Berger C., Nenov V., Machuron F., Briche M. (2023). Meta-analysis study of the effects of yeast probiotic supplementation on milk production and energy corrected milk of lactating dairy cows. Agric. Sci..

[51] Maxin G., Rulquin H., Glasser F. (2011). Response of milk fat concentration and yield to nutrient supply in dairy cows. Animal.

[52] Anadón A., Ares I., Martínez-Larrañaga M.R., Martínez M.A., Banskalieva V., Bhatt D., Bhargava N. (2019). Prebiotics and probiotics in feed and animal health. Nutraceuticals in Veterinary Medicine.

[53] Roach L. (2020). The Effect of a Seaweed Sulfated Xylorhamnoglucuronan Extract on Gut, Metabolic and Skin Health Outcomes. Ph.D. Thesis.

[54] Niederwerder M.C. (2018). Fecal microbiota transplantation as a tool to treat and reduce susceptibility to disease in animals. Vet. Immunol. Immunopathol..

[55] Rosa F., Michelotti T.C., St-Pierre B., Trevisi E., Osorio J.S. (2021). Early life fecal microbiota transplantation in neonatal dairy calves promotes growth performance and alleviates inflammation and oxidative stress during weaning. Animals.

[56] Yu H., Zhang Y., Yang D., Luo H., Zhou Y. (2025). Advances in capsule-based fecal microbiota transplantation: Clinical applications and innovations. J. Transl. Med..

[57] Malard L.A., Guisan A. (2023). Into the microbial niche. Trends Ecol. Evol..

[58] Hess M.K., Zetouni L., Hess A.S., Budel J., Dodds K.G., Henry H.M., Brauning R., McCulloch A.F., Hickey S.M., Johnson P.L. (2023). Combining host and rumen metagenome profiling for selection in sheep: Prediction of methane, feed efficiency, production, and health traits. Genet. Sel. Evol..

[59] Yan M. (2024). Elucidating the Under-Explored Genomic Diversity and Metabolic Potential of the Rumen Microbiome through Multi-Omics Approaches. Ph.D. Thesis.

[60] Krause D.O., Denman S.E., Mackie R.I., Morrison M., Rae A.L., Attwood G.T., McSweeney C.S. (2003). Opportunities to improve fiber degradation in the rumen: Microbiology, ecology, and genomics. FEMS Microbiol. Rev..

[61] Ma J., Zhang J., Guo X. (2025). Harnessing CRISPR-Cas9 for *Lactobacillus* improvement in silage production: Current knowledge and future perspectives. J. Anim. Sci. Biotechnol..

[62] Carreño E.O.B., Elghandour M.M.M.Y., De Palo P., Maggiolino A., De Angelis M., Salem A.Z.M. (2026). Nano/microencapsulation of feed additives for ruminal microbiome modulation and enteric methane mitigation in ruminants: A critical review. Front. Vet. Sci..

[63] Lv X., Qiao Z., Chen C., Hua J., Zhou C. (2025). Exploration of multi-source lignocellulose-degrading microbial resources and bioaugmentation strategies: Implications for rumen efficiency. Animals.

[64] Cho H.M., González-Ortiz G., Melo-Durán D., Heo J.M., Cordero G., Bedford M.R., Kim J.C. (2020). Stimbiotic supplementation improved performance and reduced inflammatory response via stimulating fiber fermenting microbiome in weaner pigs housed in a poor sanitary environment and fed an antibiotic-free low zinc oxide diet. PLoS ONE.

[65] Kumar S., Singh O., Kundgir S., Mahna N., Gupta M., Yadav P., Tyagi N., Samanta A.K., Tyagi A.K. (2025). Prospects of probiotics in improvement of health and productivity of dairy animals and reducing antibiotic load on the environment. Biotechnol. Environ..

[66] Mazzantini D., Calvigioni M., Celandroni F., Lupetti A., Ghelardi E. (2021). Spotlight on the compositional quality of probiotic formulations marketed worldwide. Front. Microbiol..

[67] Xiang Q., Wu X., Pan Y., Wang L., Cui C., Guo Y., Zhu L., Peng J., Wei H. (2020). Early-life intervention using fecal microbiota combined with probiotics promotes gut microbiota maturation, regulates immune system development, and alleviates weaning stress in piglets. Int. J. Mol. Sci..

[68] Kimman T.G., Smit E., Klein M.R. (2008). Evidence-based biosafety: A review of the principles and effectiveness of microbiological containment measures. Clin. Microbiol. Rev..

[69] National Academies Keck Futures Initiative (2010). Synthetic Biology: Building on Nature’s Inspiration: Interdisciplinary Research Team Summaries.

[70] Zhao Y., Zhao H., Li L., Tan J., Wang Y., Liu M., Jiang L. (2023). Multi-omics analysis reveals that the metabolite profile of raw milk is associated with dairy cows’ health status. Food Chem..

[71] Wang Z., Wang S., He Q., Yang X., Zhao B., Zhang H., Deng Y. (2025). Ecological design of high-performance synthetic microbial communities: From theoretical foundations to functional optimization. ISME Commun..

[72] Kapuscinski A.R., Letourneau D.K., Burrows B.E. (2002). Controversies in designing useful ecological assessments of genetically engineered organisms. Genetically Engineered Organisms: Assessing Environmental and Human Health Effects.

[73] Balehegn M., Duncan A., Tolera A., Ayantunde A.A., Issa S., Karimou M., Zampaligré N., André K., Gnanda I., Varijakshapanicker P. (2020). Improving adoption of technologies and interventions for increasing supply of quality livestock feed in low- and middle-income countries. Glob. Food Secur..

[74] Kebede A.B. (2009). Characterization of Milk Production Systems, Marketing and On-Farm Evaluation of the Effect of Feed Supplementation on Milk Yield and Milk Composition of Cows at Bure District, Ethiopia. Ph.D. Thesis.

[75] Tricarico J.M., Johnston J.D., Dawson K.A., Hanson K.C., McLeod K.R., Harmon D.L. (2005). The effects of an Aspergillus oryzae extract containing alpha-amylase activity on ruminal fermentation and milk production in lactating Holstein cows. Anim. Sci..

[76] Zhang H., Lu T., Guo S., He T., Shin M.K., Luo C., Tong J., Zhang Y. (2025). Rumen microbes affect the somatic cell counts of dairy cows by modulating glutathione metabolism. mSystems.

[77] Mills S., Lane J.A., Smith G.J., Grimaldi K.A., Ross R.P., Stanton C. (2019). Precision nutrition and the microbiome part II: Potential opportunities and pathways to commercialisation. Nutrients.

[78] Davies S.J., Esposito G., Villot C., Chevaux E., Raffrenato E. (2022). An evaluation of nutritional and therapeutic factors affecting pre-weaned calf health and welfare, and direct-fed microbials as a potential alternative for promoting performance: A review. Dairy.

[79] Degenshein M. (2026). Effects of Bovine-Derived Direct-Fed Microbials and Transition Milk on Dairy Calf Health Through Weaning. Master’s Thesis.

[80] Smith R.P., May H.E., AbuOun M., Stubberfield E., Gilson D., Chau K.K., Crook D.W., Shaw L.P., Read D.S., Stoesser N. (2023). A longitudinal study reveals persistence of antimicrobial resistance on livestock farms is not due to antimicrobial usage alone. Front. Microbiol..

[81] Hossein-Zadeh N.G. (2026). Advancing climate-resilient livestock systems: Next-generation emission mitigation strategies and integrated technological innovations. Vet. Anim. Sci..

[82] Chavan M.K., Dhage S.A., Gaikwad U.S., Deokar D.K., Lokhande A.T., Kamble D.K. (2024). Digital livestock farming: A review. Int. J. Adv. Biochem. Res..

[83] Li T., Ruirui Z., Zhao H., Linhuan Z., Xu G., Tongchuan Y., Wang W. (2025). Advancements in intelligent monitoring technologies for behavioral, physiological, and biomarker analysis in cattle health: A review. Agriculture.

[84] Vakulya G., Hajnal É., Udvardy P., Simon G. (2024). In-depth development of a versatile rumen bolus sensor for dairy cattle. Sensors.

[85] Silva F.G., Conceição C., Pereira A.M., Cerqueira J.L., Silva S.R. (2023). Literature review on technological applications to monitor and evaluate calves’ health and welfare. Animals.

[86] Neethirajan S., Kemp B. (2021). Digital twins in livestock farming. Animals.

[87] Garcia S.N., Osburn B.I., Cullor J.S. (2019). A one health perspective on dairy production and dairy food safety. One Health.

[88] Manono B.O. (2026). Methane emissions from livestock operations: Sources, sinks, and mitigation strategies. Methane.

[89] Wu Z., Famous M., Stoikidou T., Bowden F.E., Dominic G., Huws S.A., Godoy-Santos F., Oyama L.B. (2025). Unravelling AMR dynamics in the rumenofaecobiome: Insights, challenges and implications for One Health. Int. J. Antimicrob. Agents.

[90] López-Catalina A., Atxaerandio R., García-Rodríguez A., Goiri I., Gutierrez-Rivas M., Jiménez-Montero J.A., González-Recio O. (2021). Characterisation of the rumen resistome in Spanish dairy cattle. Anim. Microbiome.

[91] Nadi W.G., Ahmed L.I., Awad A.A.N., Taher E.M. (2024). Occurrence, antimicrobial resistance, and virulence of *Staphylococcus aureus*, *Escherichia coli*, and *Pseudomonas aeruginosa* isolated from dairy products. Int. J. Vet. Sci..

[92] Ma T., Li F., Zaheer R., McAllister T.A., Guan L.L. (2020). Host genetics affected the resistome and its expression patterns in the rumen of beef cattle raised without antibiotics used in humans. Res. Sq..

[93] Rasmussen M.A., Casey T.A. (2001). Environmental and food safety aspects of *Escherichia coli* O157:H7 infections in cattle. Crit. Rev. Microbiol..

[94] Oyama L.B., Girdwood S.E., Cookson A.R., Fernandez-Fuentes N., Privé F., Vallin H.E., Wilkinson T.J., Golyshin P.N., Golyshina O.V., Mikut R. (2017). The rumen microbiome: An underexplored resource for novel antimicrobial discovery. npj Biofilms Microbiomes.

[95] Oyama M.A., Ellenberg S.S., Shaw P.A. (2017). Clinical trials in veterinary medicine: A new era brings new challenges. J. Vet. Intern. Med..

[96] Chen C., Huang X., Fang S., Yang H., He M., Zhao Y., Huang L. (2018). Contribution of host genetics to the variation of microbial composition of cecum lumen and feces in pigs. Front. Microbiol..

[97] Schöpke K., Swalve H.H. (2016). Opportunities and challenges for small populations of dairy cattle in the era of genomics. Animal.

[98] Li Z., Wang Y., Liu J., Rawding P., Bu J., Hong S., Hu Q. (2021). Chemically and biologically engineered bacteria-based delivery systems for emerging diagnosis and advanced therapy. Adv. Mater..

[99] Mosites E., Sammons M., Otiang E., Eng A., Noecker C., Manor O., Hilton S., Thumbi S.M., Onyango C., Garland-Lewis G. (2017). Microbiome sharing between children, livestock and household surfaces in western Kenya. PLoS ONE.

[100] Ortiz-Chura A., Popova M., Morgavi D.P. (2024). Ruminant microbiome data are skewed and unFAIR, undermining their usefulness for sustainable production improvement. Anim. Microbiome.

[101] Alayande K.A., Aiyegoro O.A., Ateba C.N. (2020). Probiotics in animal husbandry: Applicability and associated risk factors. Sustainability.

[102] Kandiyal B., Gupta M., Das B. (2026). Live Biotherapeutics: Emerging Trends and Future Directions in Microbial Therapy. Prog. Mol. Biol. Transl. Sci..

[103] Malenje E.M., Missohou A., Tebug S.F., König E.Z., Jung’a J.O., Bett R.C., Marshall K. (2022). Economic analysis of smallholder dairy cattle enterprises in Senegal. Trop. Anim. Health Prod..

[104] Lerner A., Lieber A.D., Nelson-Dooley C., Leu A., Perro M., Koch G., Benzvi C., Smith J. (2026). Genetically modified microorganisms: Risks and regulatory considerations for human and environmental health. Microorganisms.

[105] Wallace R.J., Sasson G., Garnsworthy P.C., Tapio I., Gregson E., Bani P., Huhtanen P., Bayat A.R., Strozzi F., Biscarini F. (2019). A heritable subset of the core rumen microbiome dictates dairy cow productivity and methane emissions. Sci. Adv..

[106] Huws S.A., Creevey C.J., Oyama L.B., Mizrahi I., Denman S.E., Popova M., Muñoz-Tamayo R., Forano E., Waters S.M., Hess M. (2018). Addressing global ruminant agricultural challenges through understanding the rumen microbiome: Past, present, and future. Front. Microbiol..

[107] Canaveral Restrepo D. (2022). Culture and Analysis of Endometrial Cells Isolated from Dairy Heifers Compared with Postpartum Cows. Ph.D. Thesis.

[108] Valldecabres A., Anderson B., Lefler J., Marotz C., Embree M.M., Lago A. (2025). Impact of a native rumen microbe supplement provided in feed on production performance and enteric methane emissions in lactating dairy cows. J. Dairy Sci..

[109] Juliyange Alias Korale Kankanamge S.H.M. (2025). Climate Change Adaptation and Greenhouse Gas Mitigation in Pasture-Based Australian Dairy Systems: A Farming Systems Modelling Approach. Ph.D. Thesis.

[110] Li M., Wang Z., Ma Z., Wang Y., Jia H., Zhang L., Chen P., Mao Y., Yang Z. (2025). Metagenomic analysis reveals microbial drivers of heat resistance in dairy cattle. Anim. Microbiome.

[111] Lim L.W.K. (2025). CRISPR-Cas applications in extremophiles: Thermophiles, psychrophiles, halophiles, acidophiles, alkaliphiles, piezophiles, xerophiles, radiophiles and metallophiles. Total Environ. Microbiol..

[112] Kasse G.E., Cosh S.M., Humphries J., Islam M.S. (2025). Leveraging artificial intelligence for One Health: Opportunities and challenges in tackling antimicrobial resistance: Scoping review. One Health Outlook.

