# Supplementary material for: Microbiome Engineering in Dairy Cattle: A Critical Review of Strategies for Disease Resistance, Productivity, and Sustainable Farming
Source: Vet Sci. 2026 Jul 31;13(8):766. doi: 10.3390/vetsci13080766 (PMC13517236; doi:10.3390/vetsci13080766)
Supplement: Supplementary file 1 [file vetsci-13-00766-s001.zip › vetsci-4434128-supplementary.pdf]

Table S1. Complete database-specific search strategies, applied filters, and records retrieved for the systematic literature search.

Database	Full Search String	Filters / Limits Applied	Records Retrieved
PubMed	(("Dairy Cattle"[Mesh] OR dairy cattle OR dairy cow* OR bovine) AND (microbiome OR microbiota OR "rumen microbiome") AND (probiotic* OR prebiotic* OR "fecal microbiota transplantation" OR metagenomic* OR CRISPR OR "synthetic biology" OR "precision livestock farming") AND ("milk production" OR "feed efficiency" OR methane OR disease OR mastitis OR "antimicrobial resistance"))	English; 2020–2026; Journal Article, Review	720
Scopus	TITLE-ABS-KEY(("dairy cattle" OR "dairy cow*" OR bovine) AND (microbiome OR microbiota OR "rumen microbiome") AND (probiotic* OR prebiotic* OR "fecal microbiota transplantation" OR FMT OR metagenomic* OR CRISPR OR "synthetic biology" OR "precision livestock farming") AND ("milk production" OR productivity OR "feed efficiency" OR methane OR disease OR mastitis OR "antimicrobial resistance"))	English; 2020–2026; Veterinary, Agricultural & Biological Sciences	610
Web of Science	TS=(("dairy cattle" OR "dairy cow*" OR bovine) AND (microbiome OR microbiota OR "rumen microbiome") AND (probiotic* OR prebiotic* OR "fecal microbiota transplantation" OR metagenomic* OR CRISPR OR "synthetic biology" OR "precision livestock farming") AND ("milk production" OR "feed efficiency" OR methane OR disease OR mastitis OR "antimicrobial resistance"))	English; 2020–2026; Core Collection	580
Google Scholar	("dairy cattle" OR "dairy cow" OR bovine) AND (microbiome OR microbiota OR "rumen microbiome") AND (probiotic OR prebiotic OR "fecal microbiota transplantation" OR metagenomics OR CRISPR OR "precision livestock farming") AND ("milk production" OR methane OR disease OR mastitis)	English; 2020–2026; first 200 results by relevance screened	200 screened out of 15900
CAB Abstracts	((dairy cattle OR dairy cow* OR bovine) AND (microbiome OR microbiota OR "rumen microbiome") AND (probiotic* OR prebiotic* OR "fecal microbiota transplantation" OR metagenomic* OR CRISPR OR "synthetic biology") AND ("milk production" OR "feed efficiency" OR methane OR disease OR mastitis OR "antimicrobial resistance"))	English; 2020–2026; via Ovid platform	2144

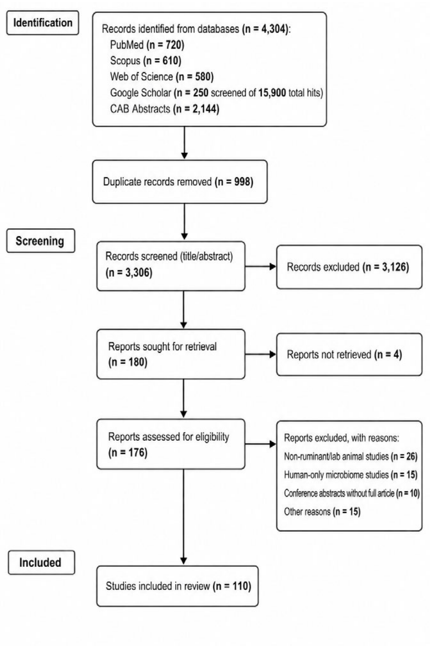

Figure S1. PRISMA 2020 flow diagram showing the identification, screening, eligibility assessment, and inclusion process of studies in the systematic review. Records were identified through database searches (PubMed, Scopus, Web of Science, Google Scholar, and CAB Abstracts), followed by duplicate removal, title/abstract screening, full-text assessment, exclusion with reasons, and final inclusion of studies. A total of 110 studies were included in the qualitative synthesis. Created in BioRender. Ashfaq, S. (2026) https://BioRender.com/jtkamoi
